# Supplementary material for: Patterns of Intron Gain and Loss in Fungi
Source: PLoS Biol. 2004 Nov 30;2(12):e422. doi: 10.1371/journal.pbio.0020422 (PMC532390; doi:10.1371/journal.pbio.0020422)
Supplement: Table S1 — Also available at http://genes.mit.edu/NielsenEtAl/. (4.3 MB ZIP). [file pbio.0020422.st001.zip › NielsenEtAl/html/143.html]

AN3857.1.NCU03763.1.MG02459.1.FG07164.1


```
 CLUSTAL W (1.82) Multiple Sequence Alignments - Introns Inserted


Sequence 1: NCU03763.1	525 aa
Sequence 2: MG02459.1	497 aa
Sequence 3: FG07164.1	502 aa
Sequence 4: AN3857.1	606 aa
Alignment Length: 782 aa
Number Identitical Residues: 154 aa
Alignment Score (without introns) 9815


MG02459.1 	MVKLEDVSSS-LLQKPS2NSNDSEIPLERRQSSYKSLKTFALDKEKQYQQQYGDMYFLRL
NCU03763.1	MVTLDEVLGSGLLQKPS2G--DAPEALQRQITNYKPLHTFKLEKTRPYQQQYADIYFLRL
FG07164.1 	MVALEEVEGS-LLQKPC2D--AAHEVPSRSASAYKPLNSFALDKQRSYKQQYGDMYFLRL
AN3857.1  	-----------------~------------------------------------------
          	                                                            

MG02459.1 	TKIKPAVEEIASTAFGDLVIGGEPARQVPRVLDVRQGELCWVAGTVYMDMPLKPSILEDV
NCU03763.1	TKIRPAVEAAASAAWEGTVLGGEKVKRVERVLDVRQGELCWVAGTVYMDMPLKPSILEDV
FG07164.1 	TKIKPAVDQVAATAWDGTTVGDEEAQRVDRVLDVRQGELCYVTGTVYMEMPLKPNILEDV
AN3857.1  	------------------------------------------------------------
          	                                                            

MG02459.1 	AKD0RWISAPTSVQKYFSKDGRDSILLEDDSGRVRLVGDILKSVVLVTGCIIAVMGTENA
NCU03763.1	SKD0RWISAPTSVGHYYSGNGEDSVMLEDDSGRIRLVGNALQNHFMVTGCIVAVMGTENA
FG07164.1 	SKD0RWISAPILTPKYFSDD--DQIMLEDESGRIRLVGDILKTVQLVTGCIVGVMGTENS
AN3857.1  	---~---------------------MLEDESGRLRLTGSKLQSVQLATGVIIAVLGTENA
          	                         :***:***:**.*. *:.  :.** *:.*:****:

MG02459.1 	SGEFEVIDLKFPDLAPQPERWSLSKP-------ADDDVEMGNGGYS-----SNRGKTAIL
NCU03763.1	NGEFEVIDLLFAELPPQPERWSLSKGK--KEADSSEDVEMMDASFSRQSHKPSSKKIALV
FG07164.1 	NGEFEIIDIKFPDLPPQPERWSLSKPVSDKSKTKDEDEEMTDVSDK----KKGNKKIAIV
AN3857.1  	NGDFEAIDIKVPDLARQPRRWERDEAK------APKEAKK--------------GKIAMV
          	.*:** **: ..:*. **.**. .:          .: :                * *::

MG02459.1 	SGLNFSSTDTSYAMELNLLLEYLLGDALDPIAQKD----ISQISRLIVAGDSIS---PDT
NCU03763.1	SGLEFSNADTSYAMELNLLLEYLLGEALDPSVQRDE---LSHITRLIIAGNSISTTAAEQ
FG07164.1 	SGLSFSGTDASYALELDLLLEYLLGEALGSSGQID----VSHISRLIIAGNSIS---TTD
AN3857.1  	SGLGITGT-SSDTLALELLTDYLLGYTGSSKGEEGSPPDASRITRLIIAGNSLG---AST
          	*** ::.: :* :: *:** :**** : ..  : ..... *:*:***:**:*:.   .  

MG02459.1 	GKPAAEEEVAG-----KKVHKKYGYDASSYNPIPSQLLDEFLSELLPTIPVTFLPGAHDP
NCU03763.1	AKPAAAFDTTTVTNKFQPQIKKYGYDSSSYNPVPSQLLDSFLSDLLPTMPVTLLPGALDP
FG07164.1 	RQPAAADETLPE----KKGQKKYGYDASTYNPLPSQLFDAFVAELLPSIPVTMLPGAQDP
AN3857.1  	IEAAPATDGVPAKK--IAHQKKYGYDASAYNASPITQLDSFLAELLPSIPITLMPGESDP
          	 :.*.  :    ..      ******:*:**. *   :* *:::***::*:*::**  **

MG02459.1 	ANASYPQQPIHPAMFTKSRTYSALPGP----------TAEPGWFDTVTNPWEGEVEGWRV
NCU03763.1	ANASYPQQPIHPAMFPQSRTYMPPPKAGAGKDRDVPGEEEPGWFDNVTNPWEGEIEGWRV
FG07164.1 	ANASYPQQPVHPAMFPAARAYTRDPAAP---------VDQPGWFDTVTNPWEAELEGWRV
AN3857.1  	ANFALPQQPIHRAMFPRSRAYCSAPPIGED-------KVEPGWFDSVTNPWEGDVEGWRF
          	** : ****:* ***. :*:*   *    .         :*****.******.::****.

MG02459.1 	LGTSGQNVDDVFKYVDSDD-------------RLGMMEAMCRWRCSAPTAPDTLW1SYPF
NCU03763.1	LGTGGQNLDDVFKYVESED-------------RLGMMEAMCRWRCVAPTAPDTLW1SYPF
FG07164.1 	LGTGGQNVDDVFKYVGSDD-------------RLGMMEAMCRWRCCAPTAPDTLW1SYPF
AN3857.1  	WGSSGQNVDDVLRYLDFADEDGNVDMNGDTEARIRVMEAMLRWRCGVPTAPDTLW1SYPF
          	 *:.***:***::*:   *.... . ...:.:*: :**** **** .******** ****

MG02459.1 	QDDDPFVMNQCPHLYFVGNQPEFGTKTITG-----TEGQ--------------EVRLITV
NCU03763.1	QEDEPFVIKECPHLFFVGCQPEFGTKVIHG-----PDGQ--------------AVRLIAV
FG07164.1 	QDDDPFVMQTCPHLYFVGNQPQFSTKVIHG-----PKGQ--------------SVRLVTV
AN3857.1  	QTHDPFVMESCPHLFFAGNQPQFKTAVIEGDLPLRPDGEDTEMGGTAQNAAVPTVRLISL
          	* .:***:: ****:*.* **:* * .* *. .  ..*:.:. ..::..:: . ***:::

MG02459.1 	PTFSAS-~---RELVLVDNETLEVTRVKISAT--~-------------------------
NCU03763.1	PSFSET-~---REVVLVDTETLDVERVKIVAE--~-------------------------
FG07164.1 	PSFSAT-~---KELVLIDTETLEVERIKISI---~-------------------------
AN3857.1  	PKFRETA1ISTSKVLLVPYSKWHVPRYHEWMKDE0EIQQATASEPLSLSEEYAMQQSWRN
          	*.*  ::  :: :::*:  .. .* * :    .. . ..::::.. : :.. : ..:  .

MG02459.1 	------------------------------------------------------------
NCU03763.1	------------------------------------------------------------
FG07164.1 	------------------------------------------------------------
AN3857.1  	DADKLTFIICLPVAPETDNGNLSLTDEDDAPTRMIGDINLFLRVEEDEEDGDEERNSSNP
          	.:.. :   . . :..:.... : :....:.:   .. .     .......... .::..

MG02459.1 	------------------------------------------------------------
NCU03763.1	------------------------------------------------------------
FG07164.1 	------------------------------------------------------------
AN3857.1  	QIIGEIELMIAEKKDQGKGFGKAALLAFLTYVIEREREVLGEFVVGDEEAKKAIGKEVKE
          	.  .. .   :........ ..::  :  :   . . .  ..   ....:..: ... ..

MG02459.1 	------------------------------------------------------------
NCU03763.1	------------------------------------------------------------
FG07164.1 	------------------------------------------------------------
AN3857.1  	LKFGALSVKIGQANERSLKLFEGLGFSKIGDSPNYFGEWELRRKELDIESENLTVDGYRE
          	 . .: : . ..:.. : .  .. . :. ..:..  .. .   .. . .:.. : ..  .

MG02459.1 	-------
NCU03763.1	-------
FG07164.1 	-------
AN3857.1  	VAYKRIA
          	 : .  :
```
